# The threat of climate change to non-dengue-endemic countries: increasing risk of dengue transmission potential using climate and non-climate datasets

**DOI:** 10.1186/s12889-019-7282-3

**Published:** 2019-07-11

**Authors:** Jung-Seok Lee, Andrew Farlow

**Affiliations:** 1 University of Oxford, Nuffield Department of Population Health, Old Road Campus, Oxford, OX3 7LF UK; 2 University of Oxford, Oxford Martin School, 34 Broad Street, Oxford, OX1 3BD UK

**Keywords:** Dengue, Dengue outbreak, Climate change, Dengue in non-dengue-endemic countries

## Abstract

**Background:**

Dengue is a major public health problem in the tropics and sub-tropics, but the disease is less known to non-dengue-endemic countries including in Northeast Asia. However, an unexpected dengue outbreak occurred in 2014 in Japan. Given that autochthonous (domestic) dengue cases had not been reported for the past 70 years in Japan, this outbreak was highly unusual and suggests that several environmental factors might have changed in a way that favors vector mosquitoes in the Northeast Asian region.

**Methods:**

A Climate Risk Factor (CRF) index, as validated in previous work, was constructed using climate and non-climate factors. This CRF index was compared to the number of reported dengue cases in Tokyo, Japan where the outbreak was observed in 2014. In order to identify high-risk areas, the CRF index was further estimated at the 5 km by 5 km resolution and mapped for Japan and South Korea.

**Results:**

The high-risk areas determined by the CRF index corresponded well to the provinces where a high number of autochthonous cases were reported during the outbreak in Japan. At the provincial-level, high-risk areas for dengue fever were the Eastern part of Tokyo and Kanakawa, the South-Eastern part of Saitama, and the North-Western part of Chiba. While a relatively small number of high-risk areas were identified in South Korea compared with Japan, the high-risk areas in South Korea include popular tourist destinations where international visitors have been increasing.

**Conclusion:**

The recent dengue outbreak in Japan may signal that the two adjacent non-dengue-endemic countries are also exposed to the risk of temporal and sporadic behavior of dengue fever. It is critical to understand potential high-risk areas for future outbreaks and to set up appropriate prevention activities at the governmental-level.

## Background

Dengue fever is a major public health concern in many parts of the tropics and sub-tropics. The disease is transmitted to humans by infective female *Aedes* mosquitoes, the two most common species being *Aedes aegypti* and *Aedes albopitus*. The epidemiology of dengue is complicated because there are four antigenically distinctive serotypes. Infection with one serotype provides life-long immunity to that specific serotype but does not confer prolonged protection against the other three serotypes [1, 2]. While clinical presentations usually exhibit mild symptoms, a potential lethal complication called severe dengue can also occur and is the main cause of hospitalization and death [3, 4].

In South Korea and Japan, a distinctive winter season exists, and temperatures fall low enough to prevent the prolonged survival of vectors, which suppresses vector-borne disease transmission. Thus, it would be difficult for these countries to be endemic either for dengue or for any other arboviral diseases. On the other hand, during the summer season, the measures of temperature, precipitation, and humidity in some parts of the two countries become similar to those observed in South- and South-East Asian countries. Despite the influx of imported dengue viruses into Japan and South Korea (i.e. carried in by international travelers), a part of the reasons why dengue infections had not been a major public-health concern in the two countries previously was because meteorological conditions were still unfavorable to the prolonged vector survival to trigger extensive vector-to-human and human-to-vector transmission. However, an unexpected dengue outbreak occurred in Japan during the summer of 2014, and 160 autochthonous (domestic) cases of dengue were reported [4, 5]. Although dengue infections had been continuously reported in Japan, an autochthonous dengue fever had not been identified for 70 years before this outbreak [5]. It was found that a subset of the domestically-infected patients visited Yoyoki Park, located in the central part of the Shinjuku-Shibuya area in Tokyo [5]. Nonetheless, it is not clear how this outbreak was triggered. Some pointed out that *Ae. albopictus*, another mosquito species which can spread dengue viruses, was expanding into the northern part of Japan including Tokyo due to climate changes, while the habitat of *Ae. aegypti* was still limited as of 2013 [5–7]. Kutsuna et al. (2015) [5] also mentioned that this epidemic period coincided with several festivals where a high number of international travelers and local people gathered, and reported that imported dengue cases were the source of the disease spread in densely-populated areas during the summer season. In South Korea, there has been no dengue outbreak observed as of March 2018. A previous study indicated that all of the identified dengue infections between 2006 and 2010 were imported cases [8]. However, *Ae. albopictus* was found in South Korea as well [8] and, in fact, two autochthonous cases were reported in 2013 and 2014 [9].

While sequential heterologous infections may lead to short-term cross-protection or more severe illness indirectly affecting the likelihood of an outbreak occurrence in dengue-endemic countries [1, 2, 10, 11], this is not the case in non-dengue-endemic countries. Rather, in Japan and South Korea—where the majority of populations are seronegative, and transmission (if any) can be sustained only in the short-run—potential conditions for a dengue epidemic would be a large number of vector mosquitoes and frequent contacts between hosts and vectors to sustain transmission [1, 2, 12]. In other words, a dengue epidemic would likely occur when vector mosquitoes increase within a short time period in a location where dengue viruses are currently circulating, and density of populations with no immunity to one of the four serotypes is high during the same period [13, 14].

The main interest of this research lies in identifying potential high-risk areas for dengue outbreaks in two non-dengue-endemic countries: Japan and South Korea. There are several factors which may cause dengue outbreaks in these countries: (1) the number of international visitors from South and South-East Asian countries where dengue is highly prevalent has been increasing, (2) domestic travelers may have been infected during their visits in dengue-endemic countries and become the source of the disease spread upon their return, (3) the climate conditions have changed over time to be more favorable to the survival of vector mosquitoes, causing a longer duration of transmission probability in the hot and humid summer season in the two countries. While the first two points have been observed in several years, these factors have not triggered dengue epidemics in Japan and South Korea. However, the recent dengue outbreak in Japan indicates that there may be increasing risks for future dengue outbreaks in both countries due to the influx of dengue viruses from overseas combined with the increase in vector population and the expansion of their habitats caused by climate change.

## Methods

A previous study indicated that the 2014 outbreak in Tokyo was related to climate conditions permitting the amplification of *Aedes* vectors and the annual peak of vectorial capacity [15]. In order to identify potential disease hotspots for dengue outbreaks, the Climate Risk Factor (CRF) index, which was previously formulated and validated against dengue incidence [2, 16], was utilized for Japan and South Korea. The CRF index was constructed based upon three climate and two non-climate datasets as shown in Table 1. Briefly, monthly climate raster files for temperature, precipitation, and humidity were obtained from January 2006 to December 2017. The night-lights data were used to estimate population density at the refined resolution. Because climate raster datasets were at relatively coarse resolutions, the raster datasets were further downscaled to a finer spatial resolution using a nearest-neighbor resampling algorithm [2, 16]. As indicated by Lee at al. (2017(a), 2017(b)) [2, 16], it was presumed that a current outbreak is a consequence of the climate conditions consistently observed during the past months, rather than single temporal values at present, and the 12-month moving average was estimated by province for each climate dataset during the study period. Temperature and humidity were positively associated with dengue incidence. However, due to the negative relationship observed between precipitation and dengue cases, the precipitation variable was reversed [2, 16, 23]. Thus all three climate datasets go towards the same underlying concept (risk for dengue fever) of the index. The datasets were standardized by subtracting the mean and by dividing by the standard deviation. The values were then averaged, and converted onto a scale from 0 to 100, with a higher number indicating higher risk. The final CRF index was adjusted by population density and elevation. The full details on the construction of the CRF index are available at [2, 16].

**Table 1:** Data description

Type	Name	Resolution^a^	Unit	Period	Sources
Climate raster	Temperature	0.5 by 0.5	Monthly	2005–2017	[17]
	Precipitation	1 by 1	Monthly	2005–2017	[18]
	Humidity	2.5 by 2.5	Monthly	2005–2017	[19]
Non-climate raster	Night lights (population density)	0.008 by 0.008	Yearly	2005 - 2013^b^	[20]
	Elevation	0.008 by 0.008	–	–	[21]
Dengue cases	Japan	By province	Weekly	2006–2016	[22]
	South Korea	By municipality	Monthly	2006–2017	[9]

^a^ All the climate raster files were resampled into 0.008 by 0.008 degree resolution by taking the nearest-neighbor assignments [2]

^b^ Due to data limitations, the night lights in 2013 was assumed to be consistent in 2014–2017, as the population density would not dramatically change during a short period of time [2]

The CRF index was first estimated at the provincial level to observe any signals prior to the epidemic in 2014 and to compare with the rapid increase in the number of autochthonous cases reported in Tokyo, Japan. Then, the CRF index was further estimated at the 5 km by 5 km resolution to identify potential disease hotspots for dengue fever in Japan and South Korea. The CRF index was categorized into 10 risk levels by cutting off every 10th value between 0 and 100. Given that there was a recent outbreak during the summer of 2014 in Tokyo, the CRF index estimated during the same epidemic period in Tokyo was used as a proxy to explain the potential climate- and non-climate-likelihood of having a dengue outbreak if dengue viruses are introduced. In other words, any geographical locations where risk levels determined by the CRF index were equal to, or higher than, the proxy category, were identified as high-risk areas for potential dengue outbreaks given the influx of dengue viruses into the areas.

### Ethics approval and consent to participate

The current study does not contain any individual persons data, thus ethics approval and consent to participate were not required.

### Permission to access data

The datasets analyzed in the current study were publically available.

## Results

**Fig. 1:**
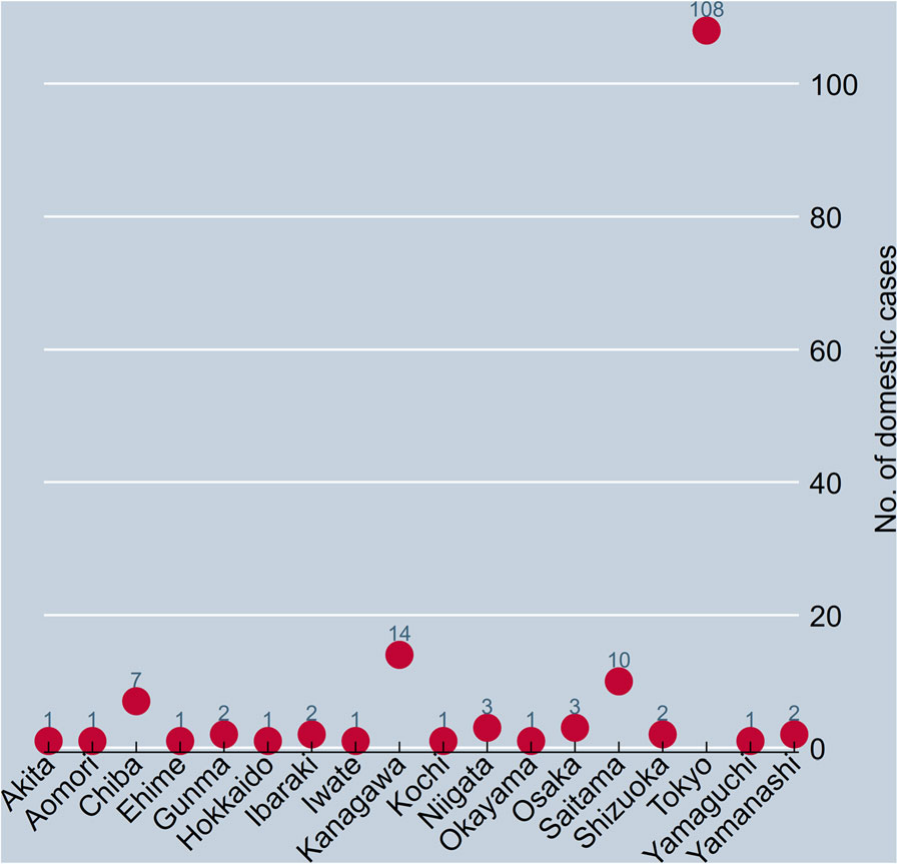
The number of domestic cases by province during 2014 in Japan

Considering that autochthonous cases had been rarely reported for the past 70 years in Japan [4, 5], the dengue outbreak which resulted in a number of domestically-infected patients in 2014 was surprising. According to the Ministry of Health in Japan [24], a total of 161 autochthonous cases were confirmed in 2014. Figure 1 shows a list of provinces where at least one domestic case was reported during 2014 in Japan. The total number of domestic cases in 2014 was by far the highest in Tokyo, with 108 confirmed episodes, followed by Kanagawa, Saitama, and Chiba provinces.

**Fig. 2:**
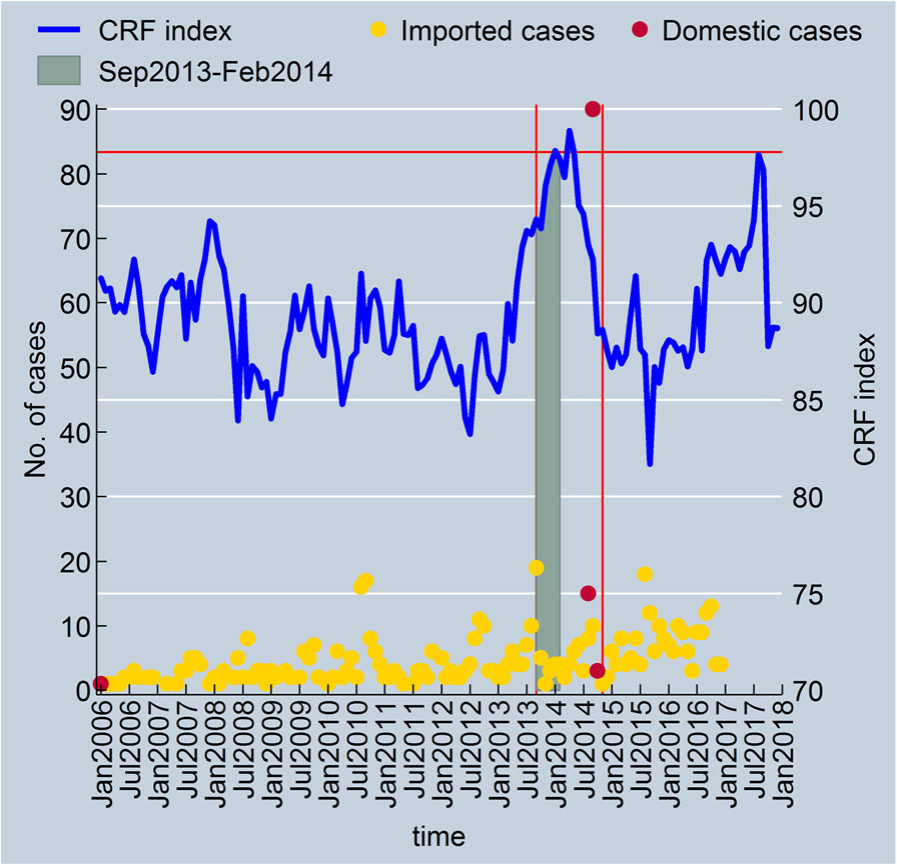
The CRF index and dengue cases over time in Tokyo, Japan

Figure 2 shows the CRF index and the number of reported dengue cases over time in Tokyo, Japan. The number of imported cases had been consistently reported from 2006 to 2016 in Tokyo, but the total number of imported cases generally remained low, with the highest number being 19 cases in September 2013. In the case of domestic episodes, no patient had been reported in Tokyo before 2014 (except one in 2006), but there was a dramatic increase in the number of autochthonous dengue patients during the summer of 2014 when the outbreak occurred. Given that the number of imported dengue cases in 2014 was not much higher than the numbers reported in other years, this epidemic was not thought to be solely caused by the influx of dengue viruses into Tokyo from travelers. Looking at the trend of the CRF index over time in Tokyo, it can be observed that the CRF index reached its peak during the same period, potentially indicating the impact of the changes in climate and non-climate factors on a large surge of infected dengue patients. The rising trend of the CRF index in 2014 was different also from other years. In Tokyo, temperature and humidity generally hit their highest levels during the summer season and go down from September to February. Though the same patterns were observed over the discrete monthly values in 2013, the CRF index did not fall even after the summer season and instead continued to rise throughout the winter until the early summer of 2014. This is because the winter of 2013 to 2014 was warmer and more humid than the winter of 2012 to 2013, leading to the increasing trend of the 12 month-moving averages for temperature and humidity. More importantly, a substantial drop in precipitation, which is negatively associated with dengue incidence, was observed throughout the first-half of 2014 and contributed to the overall increase of the CRF index. Combining all, transmission probability between hosts and vectors may have been escalated and prolonged in this densely populated city due in part to the more favorable environmental conditions for vector survival.

**Fig. 3:**
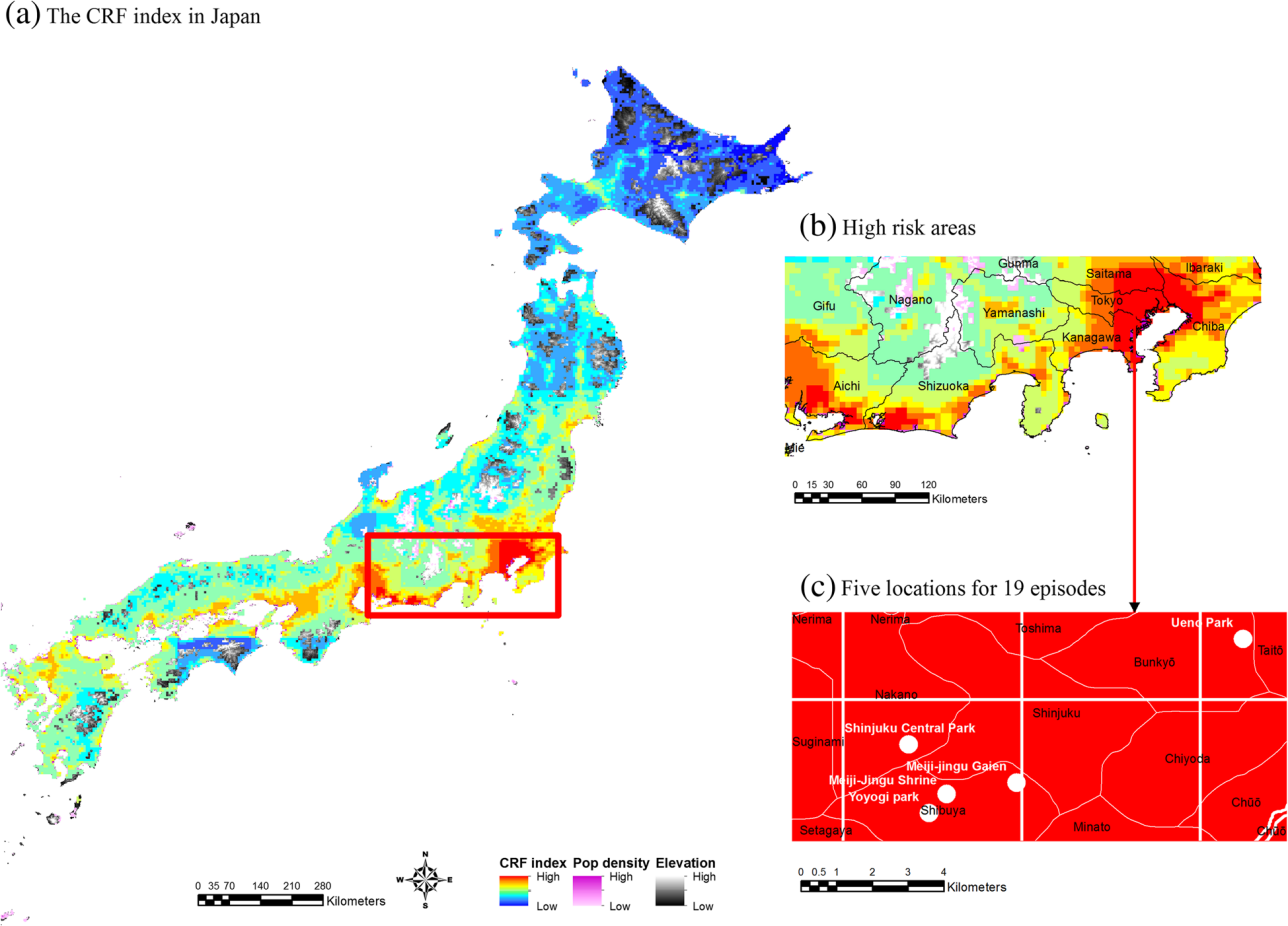
Geographical variation of the CRF index during an outbreak in Japan

The geographical variation of the CRF index was further estimated at the 5 km by 5 km resolution during the epidemic period in Japan, and is shown in Fig. 3a. At the provincial-level (Fig. 3b), high-risk areas for dengue fever were the Eastern part of Tokyo and Kanakawa, the South-Eastern part of Saitama, and the North-Western part of Chiba. These areas exactly correspond to the top four provinces where the majority of autochthonous cases were identified in Fig. 1. Small fractions of the Southern part of Shizuoka and Aichi also appear to be high-risk areas. Previously, Kutuna et al. (2015) [5] followed up on a subset of domestically-infected cases during the 2014 epidemic in Tokyo and found that 15 out of 19 patients were bitten by mosquitoes during their visits to Yoyogi Park, and the rest of the patients visited Shinjuku Central Park, Meiji-Jingu Shrine, Meiji-Jingu Gaien, and Ueno Park. The CRF index in Fig. 3c clearly indicates that all five public parks were at the greatest risk in 2014.

**Fig. 4:**
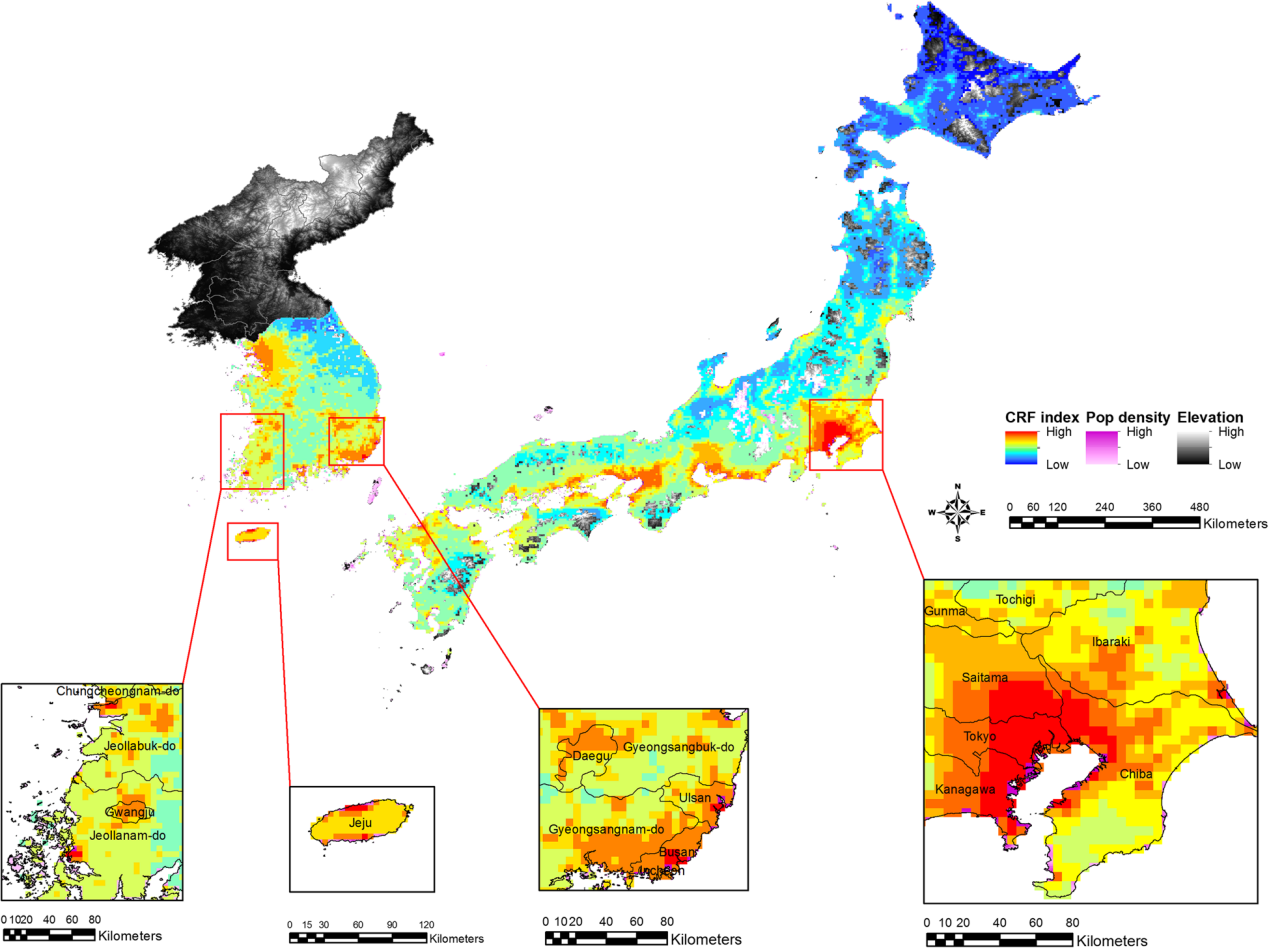
Potential disease hotspots in South Korea and Japan

Figure 4 demonstrates potential disease hotspots during the summer of 2017 using the most recent climate- and non-climate datasets. In Japan, while the Southern part of Shizuoka and Aichi provinces is no longer at high risk in 2017, the other four aforementioned provinces still appear to be high-risk areas for dengue fever. In general, the risk levels for dengue outbreaks are lower in South Korea than in Japan. Nonetheless, the following locations in South Korea were identified as high-risk areas in the case that dengue viruses start circulating: Dongnae, Yeonje, Busanjin, Suyeong, and Haeundae in Busan; the Western part of Ulsan; Gunsan; the Southern part of Muan; the Northern and Southern parts of Jeju island. In particular, both Busan and Jeju are popular tourist destinations among domestic and international travelers including those from dengue-endemic countries. It should be noted that the central and Western parts of Seoul are also at risk, although the city is not at the highest risk-level.

## Discussion

The current study investigated the potential causes of the dengue outbreak in 2014 in Japan by using the CRF index which was previously formulated in other dengue-endemic countries [2, 16]. The high-risk areas determined by the CRF index corresponded well to the provinces where a high number of autochthonous cases were reported during the outbreak in Japan. The CRF index was further estimated at a 5 km by 5 km resolution in order to identify high-risk areas for future dengue outbreaks in Japan and South Korea. In both countries, dengue fever has not been prevalent. However, the recent dengue outbreak in Japan may signal that the two adjacent non-dengue-endemic countries might also be exposed to the risk of temporal and sporadic behavior of dengue fever. Because the disease has not been widely known in both countries, it is particularly challenging to raise the awareness of the general public and among health officials, and to prepare for potential outbreaks. For example, in South Korea there was an outbreak of Middle East Respiratory Syndrome (MERS) in 2015 but, although the healthcare infrastructure was well-established in the country, not many people were aware of the disease. Furthermore, the outbreak of the disease, which originated from a Middle-Eastern country, was completely unexpected. Late diagnosis, quarantine failure of super spreaders, and poor communication by the government resulted in the failure to control the disease spread during the early phase of the disease introduction, and this put the entire society in a chaotic situation, affecting the regular routines of the general public [25]. This clearly indicates that understanding the potential risks and geographical variation of a disease are critical to minimizing the detrimental impacts of an outbreak. Even if dengue fever is not a major public health concern in both countries, the previous outbreak in Japan and the changes in environmental conditions provide the lesson that non-dengue-endemic countries can be also exposed to temporal epidemics of vector-borne diseases.

Some areas of uncertainty deserve attention. The CRF index may not be the only factor that causes dengue outbreaks. It should be noted that the CRF index itself does not take into account whether any dengue viruses are currently circulating in an area or not. In other words, even when the level of the CRF index is high, dengue infections cannot occur if there is no virus circulating within an area of interest. Furthermore, it would be rare to experience dengue outbreaks in non-dengue-endemic countries if the sources of transmission potentials were reduced by various control activities and case monitoring. For example, in 2017 there was no outbreak reported in Tokyo even though the CRF index value was relatively high. Apart from the fact that the CRF index value in 2017 was still lower than the one observed during the outbreak in 2014, this may in part have been due to the enhanced case monitoring and control activities in Japan since the 2014 outbreak. Indeed, the Ministry of Health of Japan officially announced updated guidelines for mosquito-borne infectious diseases in April 2015, after the outbreak [26]. The climate datasets were at coarse resolutions; while the datasets were resampled using the nearest neighbor assignments, the study outcomes can be further improved upon by the availability of climate datasets at much finer resolutions. Lastly, due to the limited number of observed autochthonous cases over time in these non-dengue-endemic countries, the current study took advantage of the statistical association previously validated between the CRF index and dengue incidence [2, 16]. In particular, it was shown that the rapid increase of the CRF index was highly sensitive to a dengue outbreak [16]. Nonetheless, further research is needed to understand country-specific relationships between the CRF index and dengue epidemics as more autochthonous cases are accumulated in the two countries in the future.

Given the unexpected dengue outbreak observed in Japan, this study attempted to disentangle potential causes of the epidemic by taking into account the changes in climate and non-climate patterns in Japan and South Korea. Because both countries are non-dengue-endemic, pre-emptive vaccinations may not be necessary. Rather, it is worth exploring the potential option of reactive vaccinations by planning a stockpile of a safe and effective dengue vaccine. Dengvaxia, CYD-TDV is the only available vaccine at the time of this research, but the recent reports announced by the vaccine manufacturer and by WHO indicate that vaccinating seronegative individuals with CYD-TDV may increase the risk of experiencing more severe illness when a breakthrough infection occurs after vaccination [27–29]. Thus, the use of CYD-TDV is not recommended for Japan and South Korea given that the majority of their populations are seronegatives. Instead, the two countries may consider other second-generation vaccine candidates that are currently in phase 3 trials.

## Conclusions

In sum, the CRF index estimated in the current study can be a useful tool for public health officials to identify potential disease hotspots and to prepare for effective intervention strategies, such as vector control activities, enhanced monitoring system for imported cases, and vaccination in high-risk areas.

## Data Availability

The data obtained for our study is publically available. The datasets generated and analyzed during the current study are available in the NOAA Earth System Research Laboratory, http://www.esrl.noaa.gov.

## Abbreviation

CRF: Climate Risk Factors

## References

[1] Rodriguez-Roche R, Gould EA (2013). Understanding the dengue viruses and Progress towards their control. Biomed Res Int.

[2] Lee JS, Carabali M, Lim JK, Herrera VM, Park IY, Villar L, Farlow A (2017). Early warning signal for dengue outbreaks and identification of high risk areas for dengue fever in Colombia using climate and non-climate datasets. BMC Infect Dis.

[3] Dengue and severe dengue [http://www.who.int/mediacentre/factsheets/fs117/en/].

[4] Miki S, Lee WC, Lee MJ (2017). A Comparative Study of the Trends of Imported Dengue Cases in Korea and Japan 2011–2015. J Clin Med Res.

[5] Kutsuna S, Kato Y, Moi ML, Kotaki A, Ota M, Shinohara K, Kobayashi T, Yamamoto K, Fujiya Y, Mawatari M (2015). Autochthonous dengue fever, Tokyo, Japan, 2014. Emerg Infect Dis.

[6] Nihei N, Komagata O, Mochizuki K, Kobayashi M (2014). Geospatial analysis of invasion of the Asian tiger mosquito Aedes albopictus: competition with Aedes japonicus japonicus in its northern limit area in Japan. Geospat Health.

[7] Kobayashi M, Komagata O, Yonejima M, Maekawa Y, Hirabayashi K, Hayashi T, Nihei N, Yoshida M, Tsuda Y, Sawabe K (2014). Retrospective search for dengue vector mosquito Aedes albopictus in areas visited by a German traveler who contracted dengue in Japan. Int J Infect Dis.

[8] Park JH, Lee DW (2012). Dengue fever in South Korea, 2006-2010. Emerg Infect Dis.

[9] Korea Centers for Disease Control & Prevention (2018). Infectious Disease Portal. Korea Centers for Disease Control & Prevention (KCDC).

[10] Bhoomiboonchoo P, Nisalak A, Chansatiporn N, Yoon IK, Kalayanarooj S, Thipayamongkolgul M, Endy T, Rothman AL, Green S, Srikiatkhachorn A (2015). Sequential dengue virus infections detected in active and passive surveillance programs in Thailand, 1994-2010. BMC Public Health.

[11] Halsey ES, Marks MA, Gotuzzo E, Fiestas V, Suarez L, Vargas J, Aguayo N, Madrid C, Vimos C, Kochel TJ (2012). Correlation of serotype-specific dengue virus infection with clinical manifestations. PLoS Negl Trop Dis.

[12] Transmission of the Dengue Virus [http://www.cdc.gov/dengue/epidemiology/].

[13] Barmak DH, Dorso CO, Otero M, Solari HG (2011). Dengue epidemics and human mobility. Phys Rev E Stat Nonlinear Soft Matter Phys..

[14] Louis VR, Phalkey R, Horstick O, Ratanawong P, Wilder-Smith A, Tozan Y, Dambach P (2014). Modeling tools for dengue risk mapping - a systematic review. Int J Health Geogr.

[15] Quam MB, Sessions O, Kamaraj US, Rocklov J, Wilder-Smith A (2016). Dissecting Japan's dengue outbreak in 2014. Am J Trop Med Hyg.

[16] Lee JS, Lim JK, Dang DA, Nguyen THA, Farlow A (2017). Dengue vaccine supplies under endemic and epidemic conditions in three dengue-endemic countries: Colombia, Thailand, and Vietnam. Vaccine.

[17] GHCN Gridded V2 data products from PSD [http://www.esrl.noaa.gov/psd/ ].

[18] GPCC Full Data Reanalysis Version 7.0 at 1.0°: Monthly Land-Surface Precipitation from Rain-Gauges built on GTS-based and Historic Data [http://www.esrl.noaa.gov/psd/].

[19] NCEP Reanalysis Derived data [http://www.esrl.noaa.gov/psd/].

[20] Nighttime Lights Time Series [http://ngdc.noaa.gov/].

[21] The Global Land One-kilometer Base Elevation (GLOBE) Digital Elevation Model [http://www.ngdc.noaa.gov/mgg/topo/globe.html].

[22] National Institute of Infectious Diseases (2018). Infectious diseases weekly report. National Institute of Infectious Diseases (NIID).

[23] Adde A, Roucou P, Mangeas M, Ardillon V, Desenclos JC, Rousset D, Girod R, Briolant S, Quenel P, Flamand C (2016). Predicting dengue fever outbreaks in French Guiana using climate indicators. PLoS Negl Trop Dis.

[24] Ministry of Health LaWoJ (2014). Dengue fever.

[25] Kim KH, Tandi TE, Choi JW, Moon JM, Kim MS (2017). Middle East respiratory syndrome coronavirus (MERS-CoV) outbreak in South Korea, 2015: epidemiology, characteristics and public health implications. J Hosp Infect.

[26] La W, National Institute of Infectious Diseases (2015). Guideline for mosquito-borne diseases. Ministry of Health.

[27] Pasteur S (2017). Sanofi updates information on dengue vaccine. Internet Document.

[28] World Health Organization (2017). Updated Questions and Answers related to information presented in the Sanofi Pasteur press release on 30 November 2017 with regards to the dengue vaccine Dengvaxia. Internet Document.

[29] World Health Organization (2018). Dengue vaccine: WHO position paper - September 2018. In: Weekly epidemiological record*.* World Health Organization.

